# Identification of the Hub genes and inhibitors associated with hypertension in children with obesity using WGCNA

**DOI:** 10.3389/fcvm.2026.1632570

**Published:** 2026-03-11

**Authors:** Si-mei Sun, Min Bai, Ying Liu, Qiong-yan Fang, Hai-jie Ma, Ze Yu, Fei-yue Wu

**Affiliations:** 1 Department of Pharmacy, Zhoushan Hospital, Wenzhou Medical University, Zhoushan, Zhejiang, China; 2 Department of Pediatrics, Zhoushan Women and Children Hospital, Zhoushan, Zhejiang, China; 3 Laboratory of Cytobiology and Molecular Biology, Zhoushan Hospital, Wenzhou Medical University, Zhoushan, Zhejiang, China; 4 Department of Pharmacy, Zhoushan Traditional Chinese Medicine Hospital, Zhoushan, Zhejiang, China

**Keywords:** children with obesity, HUVEC, hypertension, s100A9, WGCNA

## Abstract

**Introduction:**

Childhood obesity is an escalating public health issue linked to comorbidities such as hypertension, which poses significant cardiovascular risks. This research aims to elucidate the molecular pathways connecting obesity to hypertension in pediatric populations, with a focus on the gene S100A9 (S100 Calcium Binding Protein A9), implicated in inflammatory responses and vascular dysfunction.

**Methods:**

We analyzed gene expression data from GSE87493 dataset and performed Weighted Gene Co-expression Network Analysis (WGCNA) to identify the differentially expressed genes (DEGs) and co-expression modules associated with childhood obesity. Functional assays were conducted to investigate the role of S100A9 in vascular endothelial dysfunction. Besides, small molecule inhibitors targeting S100A9 were screened for therapeutic potential.

**Results:**

Five DEGs, including S100A9, showed distinct expression patterns in children with obesity. Mechanistically, S100A9 increases reactive oxygen species (ROS) levels and reducing Nitric oxide (NO), thereby impairing endothelial function. Small molecule inhibitor ABR-215757 (Paquinimod) was identified as a promising candidate, enhancing tube formation in the human umbilical vein endothelial cells (HUVECs) and reducing inflammatory markers.

**Conclusion:**

Targeting S100A9 may restore endothelial function and offer novel therapeutic strategies for obesity-related hypertension in children. More future research should validate these findings through *in vivo* models and clinical trials to evaluate the efficacy and safety of S100A9 inhibitors in pediatric populations.

## 1 Introduction

Obesity is a recognized risk factor for hypertension (1). Besides, hypertension constitutes one of the diagnostic criteria for metabolic syndrome, which is identified in approximately 40% of individuals who are obese (2). The incidence of hypertension among children with obesity can reach as high as 35% (3). The rise of childhood obesity has become a prominent public health concern, with a significant surge in its prevalence over recent decades. This condition transcends mere aesthetic issues, being linked to a multitude of serious health threats, including hypertension, which can have enduring implications for cardiovascular well-being.

Children who are overweight or obese are at an increased risk of developing hypertension in their adult years. The focus on preventative measures is crucial, especially for hypertension, where effective early diagnostics and biomarkers are sought. For pediatric hypertension, 24-hour ABPM is an effective diagnostic tool (4–6), and the innovative genetic methods like microarrays may help identify biomarkers.

The escalating prevalence of childhood obesity highlights the pressing necessity to unravel the intricate mechanisms that associate obesity with hypertension, as this insight could guide both preventive and therapeutic interventions. Although an array of genetic, environmental, and the lifestyle factors has been identified as contributors to the emergence of obesity in children, there remains a significant void in our comprehension of the precise molecular pathways that link obesity to the heightened susceptibility to hypertension in this at-risk demographic (7, 8).

Current investigations have revealed several genes linked to obesity; however, the specific contributions of the genes in facilitating the connection between obesity and hypertension remain inadequately examined. Among these, the S100A9 gene has attracted interest due to its role in inflammatory mechanisms and vascular impairment, both of which are important elements in the pathophysiology of hypertension. Prior research has underscored the importance of inflammatory indicators within the realm of obesity-related hypertension, yet the potential involvement of S100A9 in this dynamic is not comprehensively defined (9, 10). Consequently, there exists an urgent necessity for studies that explore the particular functions and regulatory pathways of S100A9 in relations to childhood obesity and its associated hypertension.

To address this research gap, our study employs a multifaceted approach that integrates gene expression analysis, WGCNA and functional assays. This combination of methods allows for a comprehensive exploration of gene expression profiles, co-expression networks, and identification of hub genes that may play pivotal roles in obesity-related hypertension. Such methodologies have proven advantageous in elucidating the complex biological processes and may facilitate the identification of novel therapeutic targets (11). The primary objective of this work is to elucidate the molecular mechanisms linking S100A9 to hypertension in children with obesity, thereby identifying key genes and pathways that could serve as potential intervention points.

## 2 Materials and methods

### 2.1 Data sources and gene expression profiles

We scoured the GEO database for high-throughput functional genomics research related to childhood obesity. The raw data derived from four GEO datasets included GSE87493, GSE55205, GSE69039, and GSE133786 (12–14). Statistical analysis, error identification, data refinement, and organization were conducted using the limma package, thereby enhancing data management capabilities. For the data normalization, the Robust Multi-array Average method was employed. GSE87493 represented the dataset for children with obesity, while GSE55205, GSE69039, and GSE133786 pertained to adult obesity datasets. A comprehensive analysis was performed by examining four distinct gene expression datasets.

### 2.2 Cell culture

HUVEC cells were purchased from Cell Bank of Shanghai Institutes for Biological Sciences (Shanghai, China) and these cells were identified by cell line STR. HUVEC cells were cultured in RPMI 1,640 medium supplemented with 10% FBS, 50 ng/mL VEGF, 50 ng/mL bFGF at 37 °C in an incubator with 5% CO_2_. The inhibitors ABR-215757 (No. HY-100442), ABR-215050 (No. HY-10528) and ABR-238901 (No. HY-141537) were all purchased from MCE Company (New Jersey, USA); The concentrations of the inhibitors and the incubation times are all presented in the Figure Legends.

### 2.3 Statistical analysis

GraphPad Prism 8.0 was used for data processing and statistical analysis. The results of experimental data from three independent replicates are presented as mean ± SD. Student's t-test was used for comparisons between two independent sample groups, one-way analysis of variance (ANOVA) was used for one-way comparisons among multiple groups, and two-way ANOVA was used for two-factor comparisons among multiple groups. *P* value < 0.05 was considered to indicate statistical significance. Furthermore, we performed FDR correction for comparisons of multiple genes to ensure the reliability of the overall conclusion.

### 2.4 Supplementary method

Supplementary Data provides the details on the methods for identifying DEGs, conducting functional enrichment analysis, CB-Dock2 molecular docking, performing Gene-MANIA analysis, WGCNA analysis and module identification, RNA extraction, reverse transcription and RT-qPCR, Three-dimensional culture assay (tube formation), ROS level measurement and Data collection of obese children.

## 3 Results

### 3.1 Identification of DEGs in GSE87493 dataset

The flowchart of our research design is shown in Supplementary Figure S1. First of all, samples from dataset GSE87493 were initially categorized into two groups: healthy children (*n* = 20) and those with obesity (*n* = 12), followed by an analysis of the differentially expressed genes (DEGs) within these cohorts. Employing a significance threshold of *p* < 0.05 and |log2 (fold-change)| > 1, we successfully identified the DEGs present in the GSE87493 dataset (Supplementary Figures S2A,B). A thorough GO and KEGG analysis were conducted on the DEGs derived from GSE87493. The findings indicated these genes were primarily associated with the activation of neutrophils, which play a crucial role in immune responses and neutrophil degranulation and protein targeting (Supplementary Figure S2C).

### 3.2 Identification of co-expression gene modules and hub genes in children with obesity

**Figure 1:**
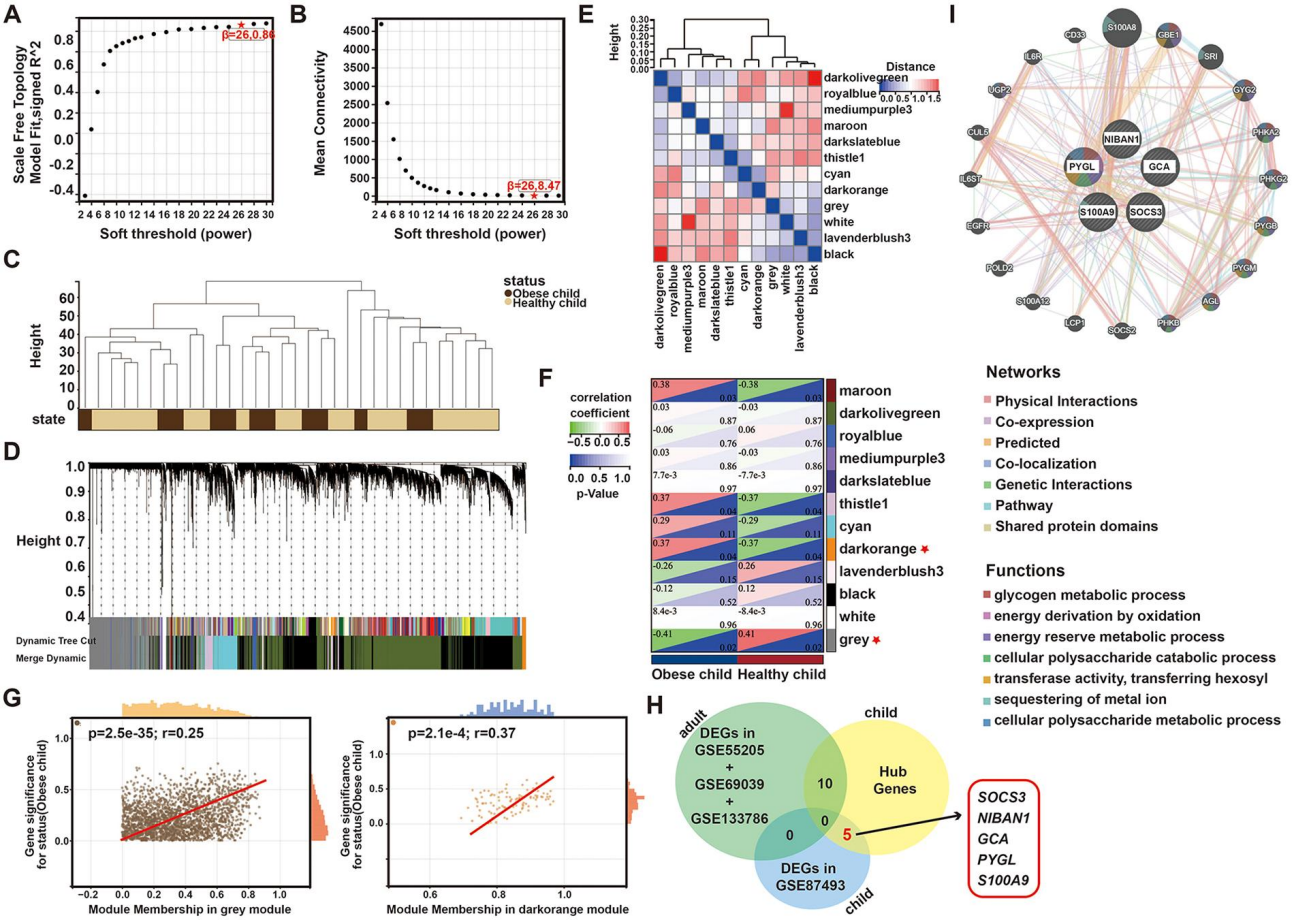
WGCNA analysis of the children with obesity via the GSE87493 dataset. **(A,B)** Analysis of network topology for various soft thresholds (*β*) **(C)** Clustering dendrogram of all samples; **(D)** The gene dendrograms obtained by average linkage hierarchical clustering; **(E)** Heatmap depicts Topological Overlap Matrix (TOM) of genes selected for weighted co-expression network analysis; **(F)** Module-trait associations; **(G)** Correlation analysis of modules with Obesity status; **(H)** Venn diagram of key module genes vs. DEGs; **(I)** Gene-MANIA was used to analyze the function and correlation of hub 5 genes of children with obesity.

We employed Weighted WGCNA to discern co-expression gene modules within the dataset of children with obesity, GSE87493. With the soft threshold power set at 26, a scale independence of 0.86 was achieved (Figures 1A,B). The samples from the two datasets were categorized into the healthy children group and the obesity children group, with no outliers identified (Figure 1C). Hierarchical clustering and dynamic branch cutting methods were applied to gene dendrogram, when the cut height was established at 0.25 and the minimum module size was set to 30, twelve distinct co-expression modules were derived through the dynamic tree cutting (Figures 1D,E). Subsequently, correlation analyses of each module with the clinical traits were conducted. The darkorange module exhibited the strongest positive correlation with obesity in children, while the grey module demonstrated the most significant negative correlation with obesity in children (Figure 1F). Furthermore, correlation analysis between MM and GS indicated that these genes were highly correlated with both modules we selected (Figure 1G and Supplementary Figure S3A).

Furthermore, we simultaneously analyzed three datasets of the adult obesity (GSE55205, GSE69039 and GSE133786) (Supplementary Figure S4A–C). Meanwhile, based on the DEGs obtained previously between the children with obesity and healthy children, the Venn analysis revealed that five of them were only present in children with obesity, including *SOCS3*, *NIBAN1*, *GCA*, *PYGL* & *S100A9* (Figure 1H). Finally, The Gene-MANIA analysis revealed *PYGL* had the most interaction among the five genes involved in a variety of cell signal transduction, including glycogen metabolic process and energy derivation through oxidation (Figure 1I). Besides, except for *SOCS3*, the other 4 genes were all upregulated in children with obesity (Supplementary Figure S3B).

We recognize that *PYGL* (hepatic glycogen phosphorylase), a crucial enzyme in glycolysis and glycogenolysis, facilitates lipogenesis by augmenting gluconeogenesis and the accumulation of acetyl-CoA, while simultaneously suppressing fatty acid *β*-oxidation. However, this study aims to investigate the risk of hypertension in children with obesity. Consequently, beyond *PYGL*, we have also observed the aberrant expression of *S100A9*. As a protein associated with inflammation, S100A9 exhibits multifaceted connections with the progression of obesity and hypertension.

To more accurately explore the core genes of this study, we first collected 50 blood samples from obese hypertensive children in Zhoushan area and 50 healthy children as the control group. Then, transcriptome sequencing was conducted, followed by PCA analysis, data collation and signaling pathway analysis (Supplementary Figures S5A–C). Finally, through qPCR verification, it was found that in the S100A family genes, Only S100A9 was significantly upregulated in obese children, and S100A8 also slightly increased. There were no significant changes in other family genes (Supplementary Figure S5D). In addition, the expression value of S100A9 in children with higher blood pressure was also significantly higher than that in the relatively hypotensive group, and S100A9 showed a positive correlation with the blood pressure values of these obese children (Supplementary Figures S5E,F). More importantly, there were no significant differences in the other 4 hub genes between obese and hypertensive children and healthy children that we collected (Supplementary Figure S5G).

S100A8/A9, as a damage-associated molecular pattern (DAMP), mainly exerts its effect by binding to Toll-like receptor 4 (TLR4) and receptor for advanced glycation end products (RAGE) (15). This combination triggers downstream signal transduction, leading to the upregulation of NADPH oxidase (Nox) expression and thereby promoting the massive generation of reactive oxygen species (ROS). Meanwhile, the activation of TLR4 will further promote the initiation of the NLRP3 inflammasome (16). ROS induced by S100A8/A9 is a key link in the activation of the NLRP3 inflammasome. The activated NLRP3 inflammasome promotes the activation of caspase-1, and then cleaps pro-IL-1β to produce mature IL-1β (17, 18). The released IL-1β can further enhance inflammatory response via autocrine/bypass circulation, which may be an important inflammatory pathway mechanism promoting the development of hypertension in children (18). The S100A8/A9- NLRP3-IL-1β axis has been proven to promote vascular inflammation and endothelial dysfunction in a variety of cardiovascular diseases (16, 18). A persistent low-grade inflammatory state may lead to vascular remodeling, which is regarded as an important mechanism in the development of hypertension (19). In addition, S100A8/A9 upregulates the expression of VCAM-1/ICAM-1, fostering the infiltration of monocytes into the vascular wall, and promotes collagen deposition, resulting in hardening of arterial walls.

Therefore, drawing from above findings and existing literature, we hypothesize that *S100A9* serves as a pivotal gene regulating hypertension in children with obesity.

### 3.3 Screening of small molecule inhibitors targeting S100A9

**Figure 2:**
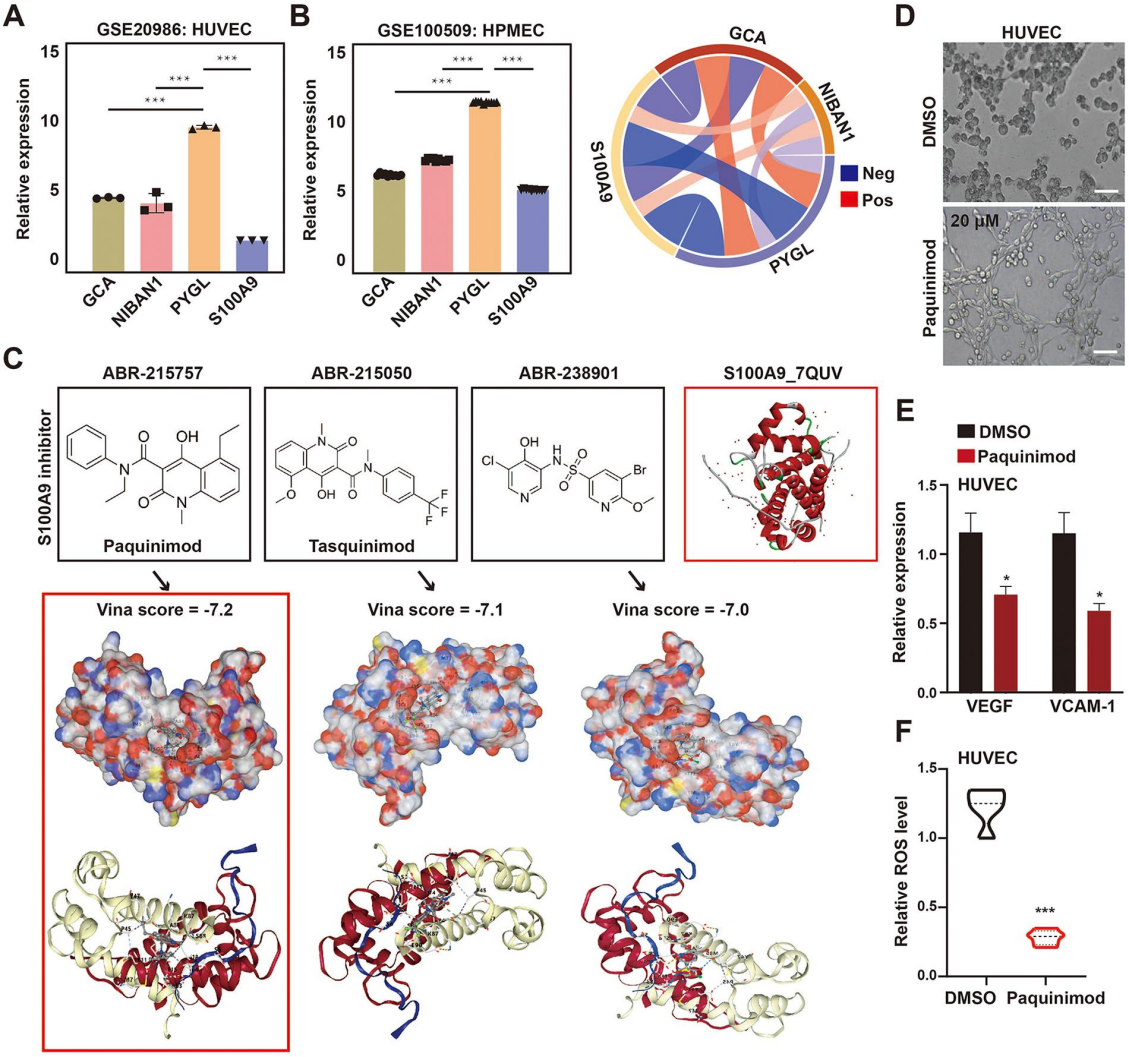
Screening and functional verification of small molecule inhibitors targeting S100A9. **(A,B)** Analysis of the expression of NIBAN1, GCA, PYGL & S100A9 genes in GSE20986 and GSE100509 datasets; **(C)** The structures and molecular docking results of S100A9 protein and three small molecule inhibitors targeting S100A9 protein were presented; **(D)** The tube formation results of HUVEC cells after the use of Paquinimod; **(E)** The expression of VEGF and VCAM-1 in HUVEC cells after Paquinimod treatment was detected by RT-qPCR; **(F)** Paquinimod significantly inhibited ROS production in HUVEC cells. These results are presented as the means ± SD of values obtained in three independent experiments, *n* = 3, *P < 0.05; **P < 0.01; ***P < 0.001.

Firstly, through the GEO datasets (GSE20986 and GSE100509) of two groups of the human vascular endothelial cells (HUVEC and HPMEC), we confirmed that all 4 genes were expressed (Figures 2A,B). Then, for S100A9 protein, we initially screened three commercial small molecule inhibitors of S100A9 (ABR-215757, ABR-215050 and ABR-238901), and their structures are shown in Figure 2C. In addition, the characteristics and application studies of these inhibitors are presented in the Supplementary Table.

Subsequently, through the CB-dock2 molecular docking website, we first identified the five pockets of S100A9 protein (Supplementary Figure S6A). These small molecules respectively docked with S100A9 protein. Results showed that all the small molecules were embedded in the C3 pocket. Meanwhile, the vina scores (VS) were −7.2, −7.1 and −7.0, respectively (Figure 2C & Supplementary Figures 6B, 7A,B). More importantly, Paquinimod can significantly reduce the protein level of S100A9, while the other two inhibitors have no obvious effect (Supplementary Figures S8A,B). Therefore, we selected ABR-215757, which has the highest binding efficiency with S100A9 protein, as the small molecule for the subsequent research for experimental study.

We first confirmed S100A9 inhibitor Paquinimod (ABR-215757) increased tube formation junctions of the channels formed in HUVEC cells, compared with the control group, and other two inhibitors did not show the clear trend (Figure 2D and Supplementary Figure S8C). These data indicated that S100A9 inhibited generation of tubular structures in HUVEC cells and inhibits cell migration, while Paquinimod can reverse this phenomenon. At the same time, Paquinimod can significantly inhibit the mRNA levels of adhesion molecules VEGF and VCAM-1, and reduce intracellular ROS (Figures 2E,F).

Furthermore, to verify the specificity of Paquinimod, we first conducted an evolutionary tree analysis on the S100A family proteins and discovered that S100A9 and S100A8 had the highest homology and sequence similarity (Supplementary Figure S8D). Therefore, next, we examined the effect of Paquinimod on S100A8 protein level. Results showed that neither Paquinimod nor the other two S100A9 inhibitors can affect the protein level of S100A8 (Supplementary Figure S8E). Moreover, both molecular docking and qPCR analyses indicated that Paquinimod had no significant interaction with other proteins of the S100A family and could not affect their mrna expression levels (Supplementary Figure S9A,B). These findings also indicated Paquinimod reduced inflammatory cell infiltration, increases the vasodilation and restores endothelial cell function.

## 4 Discussion

Childhood obesity is a growing public health issue linked to serious health outcomes like hypertension in youth. Its origins are complex, involving genetic, environmental, and lifestyle factors, leading to increased prevalence. Children with obesity face immediate and long-term health challenges, including cardiovascular diseases, emphasizing the need for effective prevention and intervention.

This study explores how the S100A9 gene connects obesity and hypertension in children, identifying S100A9 as potential biomarker and mechanistic mediator. Findings show S100A9 affects the inflammation and vascular dysfunction, crucial for understanding obesity-related hypertension, and highlights its role in activating NADPH oxidase, increasing ROS, and decreasing NO, suggesting it as a target for treatment.

S100A9 is a calcium-binding protein, belonging to the damage-associated molecular pattern (DAMP) protein category, and is widely involved in inflammatory responses and fibrotic processes. In the context of childhood hypertension, the potential of S100A9 as a biomarker is mainly reflected in its association with inflammation and immune activation. Our study suggested that S100A9 is positively correlated with hypertension in obese children, which is consistent with the reports in the literature that S100A9 serves as an inflammatory marker in various diseases (20, 21). For instance, in cardiovascular diseases, S100A9 is identified as a key biomarker, involved in pathological processes such as inflammation, oxidative stress and fibrosis, which are also common in the pathogenesis of hypertension (21).

Compared with the existing biomarkers of childhood hypertension [C-reactive protein (CRP), renin, aldosterone], S100A9 may have unique advantages. Traditional biomarkers such as CRP are mainly used to assess systemic inflammation, but S100A9 is more specifically associated with the activation of neutrophils and macrophages (20, 22). For example, in obesity-related diseases, the level of S100A9 is associated with metabolic disorders and inflammatory states, while existing markers may lack sensitivity to early immune dysregulation. Specifically, S100A9 has shown association with hyperglycemia and insulin resistance in the diabetes and obesity models, while in children with hypertension, neutrophil activation as a major feature may make S100A9 more specific in identifying obesity-related hypertension subgroups (23, 24). In terms of clinical diagnostic utility, the S100A9 may achieve minimally invasive screening by testing serum or blood samples, and at the same time, it can be combined with other markers (such as BMI) to improve accuracy (21, 24).

The investigation reveals how childhood obesity affects immune functionality through neutrophil degranulation pathways, suggesting persistent inflammation leads to comorbidities like hypertension. Understanding S100A9 interactions may clarify obesity's immunological impact, aiding in developing immune modulators for better obesity management. Limitations include small sample size, lack of clinical validation, and potential batch effects, affecting the robustness of findings.

Paquinimod is an S100A9 inhibitor that has shown therapeutic potential in multiple studies, but its safety data in the pediatric population is limited. According to the literature, the safety and pharmacokinetics of Paquinimod have been confirmed in adult clinical trials (IPF and diabetes) (22, 23). For instance, in idiopathic pulmonary fibrosis model, Paquinimod improved the pathological changes of fibrosis and no serious adverse events were reported (22). Similarly, in retinopathy studies, Paquinimod inhibited S100A9-driven NLRP3 inflammasome activation *in vitro* (25). However, children's responses to therapeutic interventions may differ from those of adults, mainly because their developing immune systems and organs are more susceptible to interference. Potential developmental effects may include unknown effects on neural development or bone growth, as S100A9 plays a role in neuro-inflammation (26), but there is a lack of long-term follow-up data for pediatric populations. Subsequent clinical applications require a large amount of follow-up data from specific populations as a basis before conducting *in vivo* studies on children.

In terms of long-term consequences, S100A9 inhibition may regulate TLR4 pathway (20). Age-appropriate dosing strategies should be based on pediatric pharmacological principles, such as adjusting the dosage according to body weight or body surface area, similar to the application of other immunomodulators in children (22). Therefore, targeted clinical trials need to be conducted in children to evaluate safety of low-dose initiation and progressive administration (27). Overall, the safety of Paquinimod in adults provides a basis for pediatric application, but the individual differences and developmental stage-specific risks need to be carefully evaluated (21, 23).

Our research has found that neutrophil activation is the main immune feature in obese children with hypertension, which is consistent with the S100A9-mediated inflammatory pathway in the literature. S100A9 drives the inflammatory response by activating Toll-like receptor 4 (TLR4) and NLRP3 inflammosomes, which has been confirmed in various diseases (20, 22, 25). Among the existing mechanisms of pediatric hypertension, the traditional pathways mainly involve the activation of the renin-angiotensin system (RAS), sympathetic hyperactivity or endothelial dysfunction (21, 28). In contrast, the neutrophil activation pathway may represent a novel mechanism that more directly links obesity-related metabolic disorders and the development of hypertension. Specifically, in obesity models, upregulation of S100A9 leads to energy metabolism disorders and increased inflammation (24). Our research shows that this pathway is prominent in childhood hypertension, suggesting it may be particularly targeted at obesity-related hypertension, as S100A9 is highly expressed in both obese and diabetic states (23, 24). However, S100A9 also plays a role in non-obesity-related diseases (such as myocardial infarction), indicating that it may be involved in a broader range of inflammatory responses, not limited to obesity (11, 27). For instance, in cardiovascular diseases, S100A9 promotes fibrosis and inflammation, processes that are common in various subtypes of hypertension (21, 28). Therefore, the patient population for targeted S100A9 inhibition therapy may include children with obesity-related hypertension, but it may also extend to other cases of inflammation-driven hypertension (20, 27). This distinction is crucial for determining the treatment strategy: if neutrophil activation is only targeted at obesity, the treatment may focus on metabolic intervention; if the response is widespread, the S100A9 inhibitor (Paquinimod) may become a supplement to the current pediatric hypertension treatment, especially for patients who do not respond to traditional therapies. At present, the field of pediatric hypertension treatment lacks options targeting immune regulation. Therefore, approach discovered in this study may fill this gap by inhibiting S100A9 to alleviate the progression of inflammatory hypertension.

The sample size of the GSE87493 dataset used in this study is relatively limited, which is a key limitation of this research. The stability of module detection in WGCNA, the estimation of network topological parameters, and the identification of hub genes all largely depend on the sample size. A smaller sample size may increase statistical variation, resulting in less clear boundaries for certain co-expression modules or less stable rankings and connections for some hub genes. Although S100A9 showed relatively high module affiliation and connectivity in the analysis, its absolute strength of “hub” status in small sample setting may need to be interpreted with caution. It may represent the most prominent association signal in the current dataset, but its network centrality may slightly change in a larger sample. The smaller sample size may limit our ability to detect weak but biologically significant co-expression patterns, meaning that some genes or modules related to obesity-induced hypertension may not be captured by the current analysis.

To compensate for this deficiency, we do not merely rely on the results of WGCNA. The selected core regulatory genes all come from the intersection of DEGs and key modules, which enhances their biological relevance. More importantly, we conducted the independent functional experimental verification of S100A9 (in the endothelial cell function experiment), providing functional-level support for the key role of S100A9 in endothelial dysfunction. At the same time, we referred to existing literature in the discussion, pointing out the role of S100A9 in adult inflammation and cardiovascular diseases, which provides indirect biological rationality support for our findings in the pediatric population.

In this study, the HUVECs used as an *in vitro* primary endothelial cell model, although it is a classic tool for studying endothelial function, has inherent simplification. It cannot fully simulate the complexity of the vascular environment in children's bodies and lacks the interactions among various cell types (such as smooth muscle cells, immune cells) that exist in the body. Therefore, the effects of S100A9 on endothelial function observed in the HUVECs experiments and the protective effect of Paquinimod provide strong “principle verification” for mechanism hypothesis, but they are not directly equivalent to the therapeutic effects in the children with hypertension. Therefore, our findings require further experiments, including animal models and a large number of clinical studies, for validation.

Furthermore, one of the core early events related to childhood obesity-induced hypertension is vascular endothelial dysfunction. HUVECs are the most commonly used and mature models for studying the proliferation, migration, apoptosis, permeability, and nitric oxide (NO) signaling pathways of endothelial cells (29). Therefore, using it to investigate whether S100A9 directly impairs endothelial cell function is relatively appropriate. Meanwhile, although HUVECs are derived from umbilical cords, the basic endothelial cell biological characteristics they exhibit (forming tubular structures and responding to inflammatory factors) are highly conserved in vascular endothelial cells in children.

Therefore, based on the above research, we clearly state that the main value of this study lies in: (1) By combining bioinformatics with experiments, S100A9 has been identified as a potential key molecular node connecting childhood obesity and endothelial dysfunction; (2) The feasibility of targeting and inhibiting S100A9 (using Paquinimod) to improve endothelial function has been verified at the cellular level. This provides important preclinical evidence for “targeting S100A9 may become a therapeutic strategy”, but its ultimate clinical applicability depends on future evaluations of its efficacy, safety, and pediatric-specific pharmacokinetics in animal models and well-designed clinical trials.

In conclusion, this study reveals key molecular mechanisms and pathways linked to hypertension in children with obesity, emphasizing *S100A9* as a critical mediator of vascular dysfunction and inflammation, and a potential biomarker and mechanistic mediator. It identifies important genes and interactions, suggesting future targeted interventions to reduce hypertension risk in this group, with further research (including a broader collection of clinical data and animal model experiments) needed to validate these findings and their implications.

## 5 Conclusions

In summary, this research aims to bridge the existing knowledge gap regarding the molecular mechanisms underlying childhood obesity and hypertension, with a specific focus on the role of S100A9. We seek to uncover the intricate relationships between gene expression, inflammatory responses, and vascular health in children with obesity via leveraging advanced analytical techniques, ultimately, the findings from this study may contribute to the development of targeted therapeutic strategies aimed at mitigating the risks of hypertension in this population.

## Data Availability

The datasets presented in this study can be found in online repositories. The names of the repository/repositories and accession number(s) can be found in the article/Supplementary Material.
