# Supplementary material for: Identification of the Hub genes and inhibitors associated with hypertension in children with obesity using WGCNA
Source: Front Cardiovasc Med. 2026 Mar 11;13:1632570. doi: 10.3389/fcvm.2026.1632570 (PMC13013500; doi:10.3389/fcvm.2026.1632570)
Supplement: Supplementary file 1 [file Datasheet1.pdf]

STR genotype test report

Sample Name: HUVEC

Detection method: DNA was extracted using Axygen's genome extraction kit, amplified using the 20-STR amplification protocol, and STR loci and the sex gene Amelogenin were detected on the ABI 3730XL genetic analyzer.

Test result: The DNA typing of this cell strain found a completely matching cell line in the cell line search. The DSMZ database shows that the cell name is HUVEC and the cell number corresponds to CRL-1730. No polyalleles were found in this cell line in this test. Cell matching value: 1.0.

STR database comparison and analysis: The genotyping results of the STR loci and Amelogenin loci of the cells to be tested were compared with the STR data of 2455 cell lines included in the ExPASY, ATCC, DSMZ, JCRB and RIKEN databases. If the cells to be tested are not included in the above cell banks or are newly established cell lines, they cannot be compared. Users need to compare them with other databases based on the cell typing results by themselves.

分型结果:

Loci	STR information of the submitted cells for			STR information of cells in the cell bank		
	Cell name for examination: HUVEC			Cell bank cell name: HUV-EC		
	Allele1	Allele2	Allele3	Allele1	Allele2	Allele3
D5S818	11	12		11	12	
D13S317	9	11		9	11	
D7S820	8	12		8	12	
D16S539	11	12		11	12	
VWA	16	16		16	16	
TH01	6	9.3		6	9.3	
AMEL	X	X		X	X	
TPOX	8	11		8	11	
CSF1PO	11	12		11	12	
D12S391	17	17				
FGA	21	23				
D2S1338	18	22				
D21S11	28	31				
D18S51	13	17				
D8S1179	14	16				
D3S1358	16	16				
D6S1043	12	18				

PENTAE	7	13				
D19S433	12	13				
PENTAD	12	13				
D1S1656	12	17.3				

Typing spectrum：

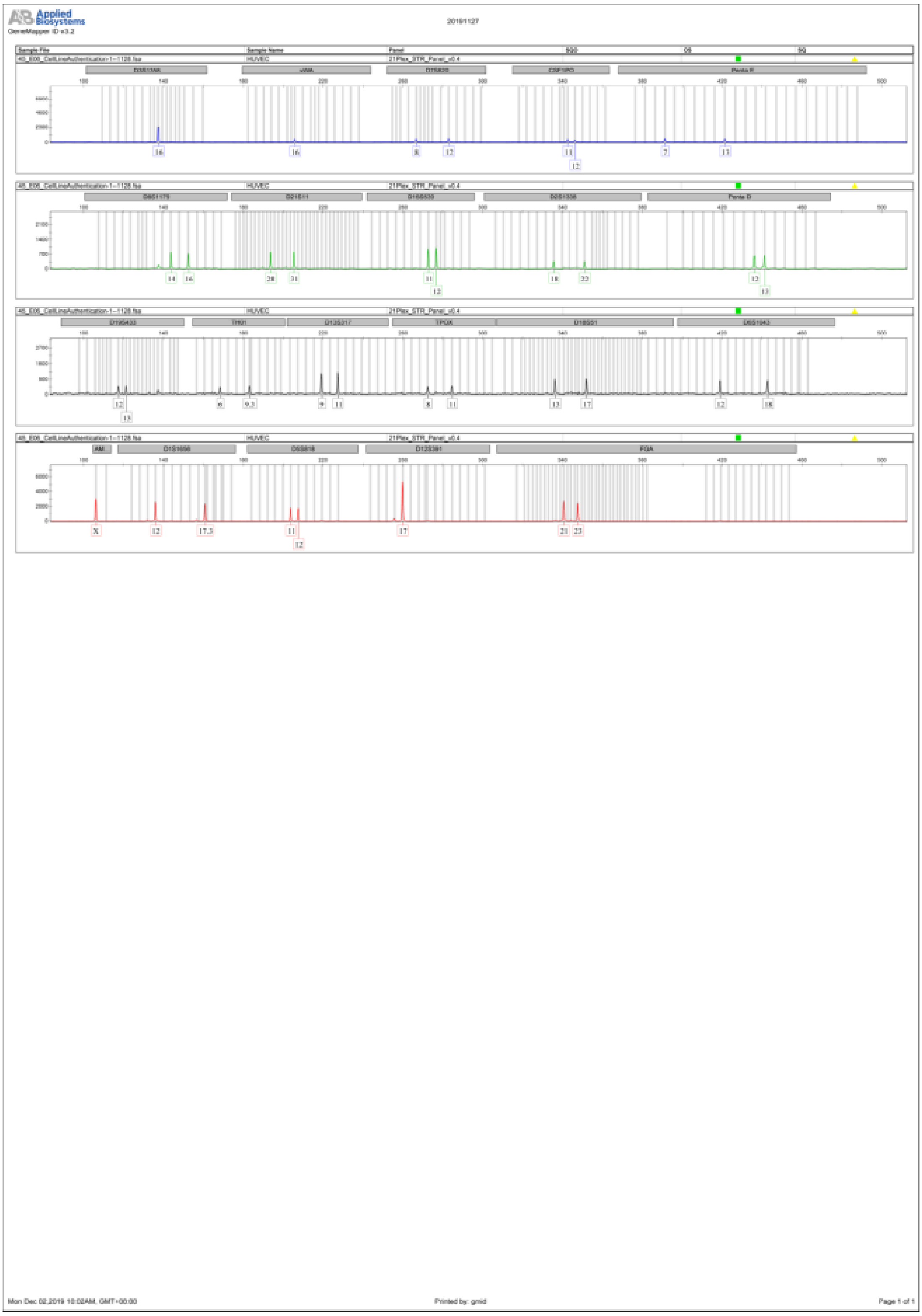

Remarks：

1. According to the STR identification standards for cells set by the International Council for Cell Identification (ICLAC), a matching degree of ≥80% is considered correct for the cell line, while a matching degree of < 80% indicates that the origin of the cell line needs to be suspected;

2. The true PCR bands of the effective peak positions in the spectrum, small peaks and non-specific bands are ignored in the calculation;

3. National Experimental Cell Resource Sharing Platform, database entry: http://www.cellresource.cn /.
