# Supplementary material for: Identification of the Hub genes and inhibitors associated with hypertension in children with obesity using WGCNA
Source: Front Cardiovasc Med. 2026 Mar 11;13:1632570. doi: 10.3389/fcvm.2026.1632570 (PMC13013500; doi:10.3389/fcvm.2026.1632570)
Supplement: Supplementary file 2 [file Datasheet2.docx]

**Supplementary Figures**

**Supplementary Figure 1**

**
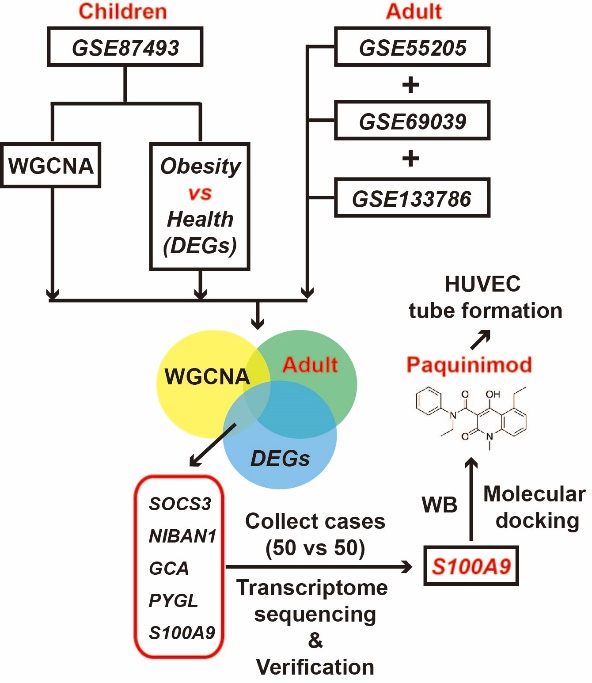
**

**Supplementary Figure 1** A flowchart of the study design.

**Supplementary Figure 2**

**
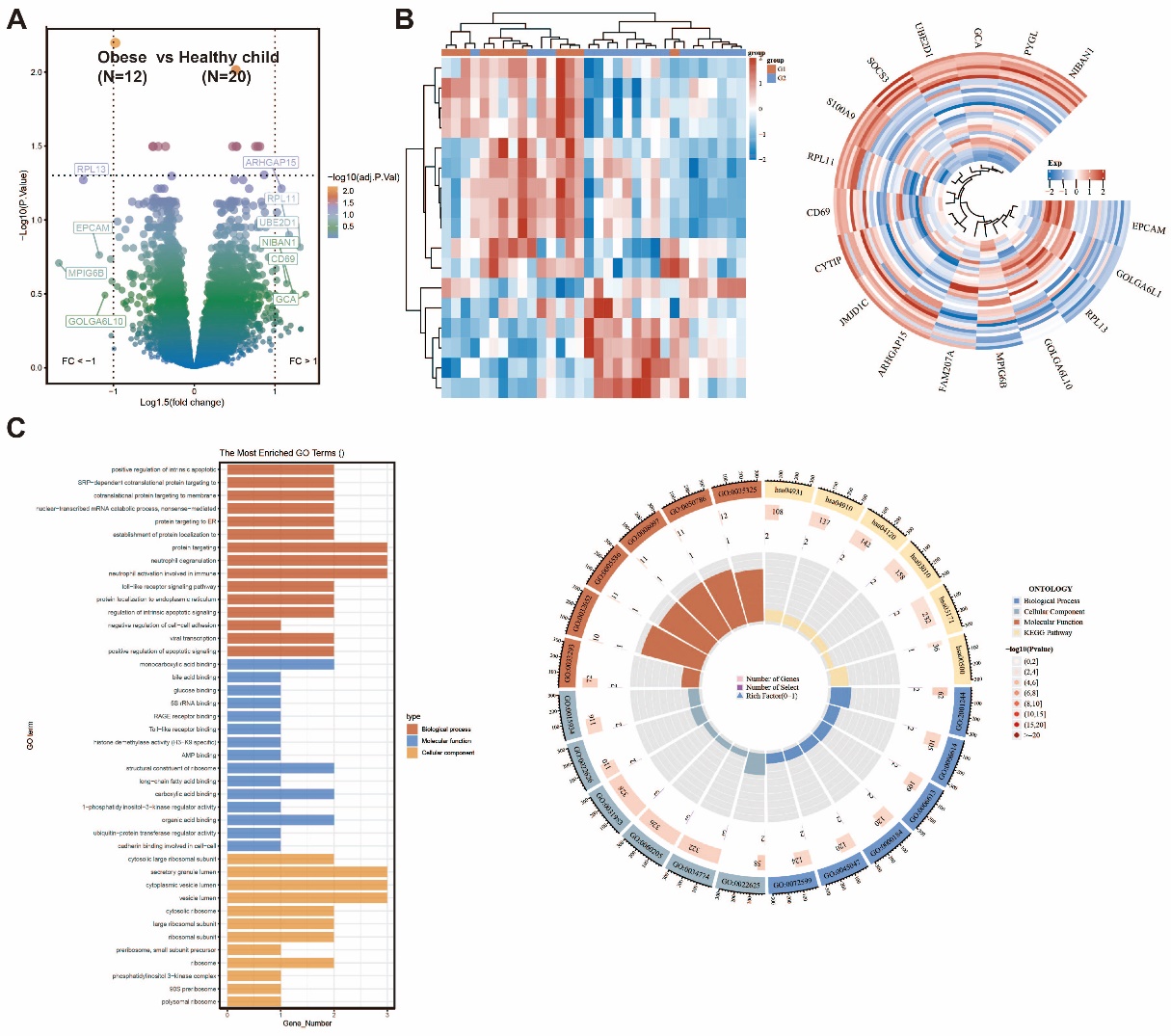
**

**Supplementary Figure 2 (A, B)** Volcano and heat-map of differential genes in **GSE87493** dataset **(obese children, N=14 vs Healthy children, N=20)**; **(C)** Functional enrichment analysis included KEGG pathway enrichment and GO term enrichment results of differential genes.

**Supplementary Figure 3**


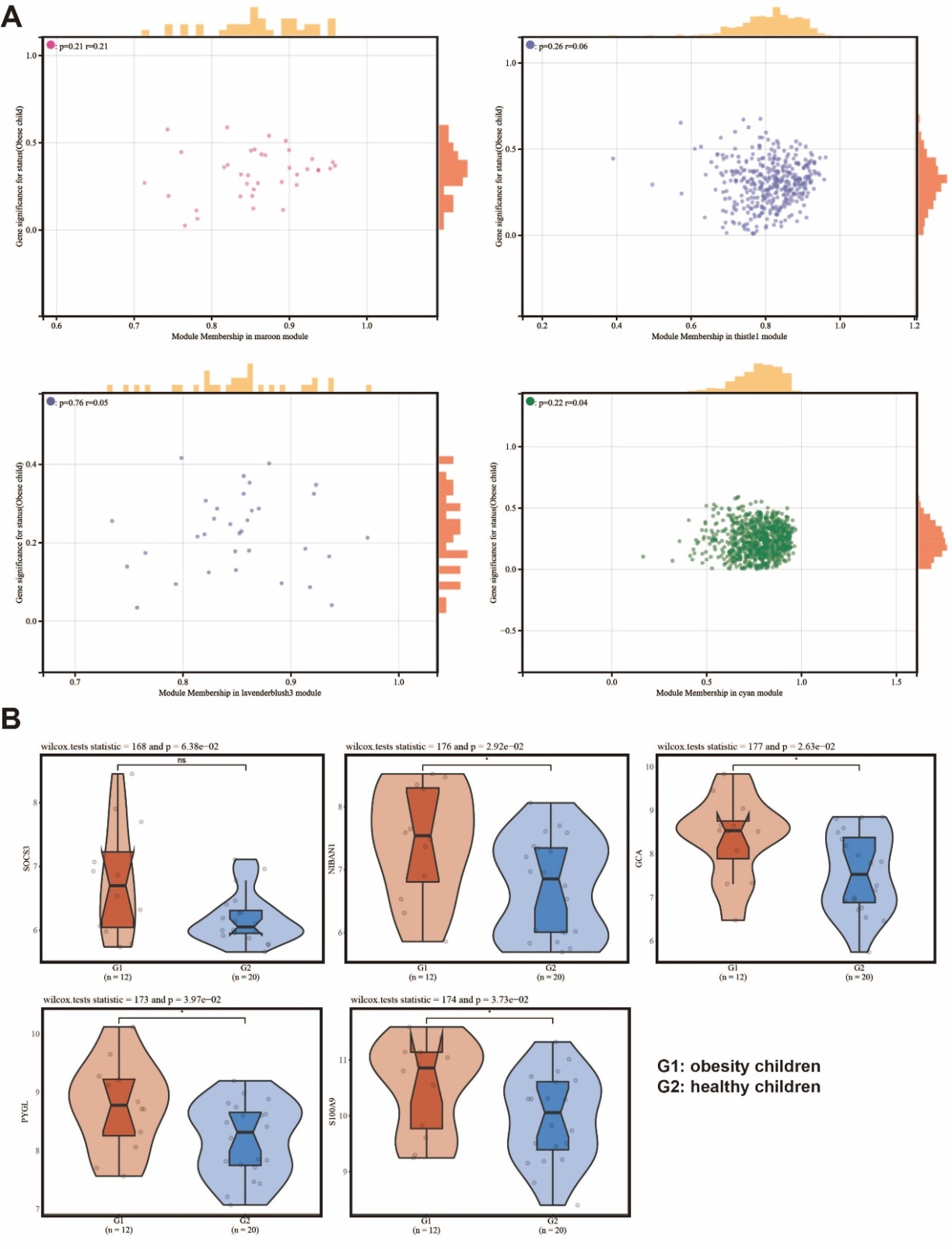


**Supplementary Figure 3 (A)** Correlation analysis of modules (maroon, thistle1, lavenderblush3 and cyan) with Obesity status; **(B)** Differential expression analysis of ***SOCS3, NIBAN1, GCA, PYGL* & *S100A9*** in the obesity children group and healthy children group of the GSE87493 dataset.

**Supplementary Figure 4**


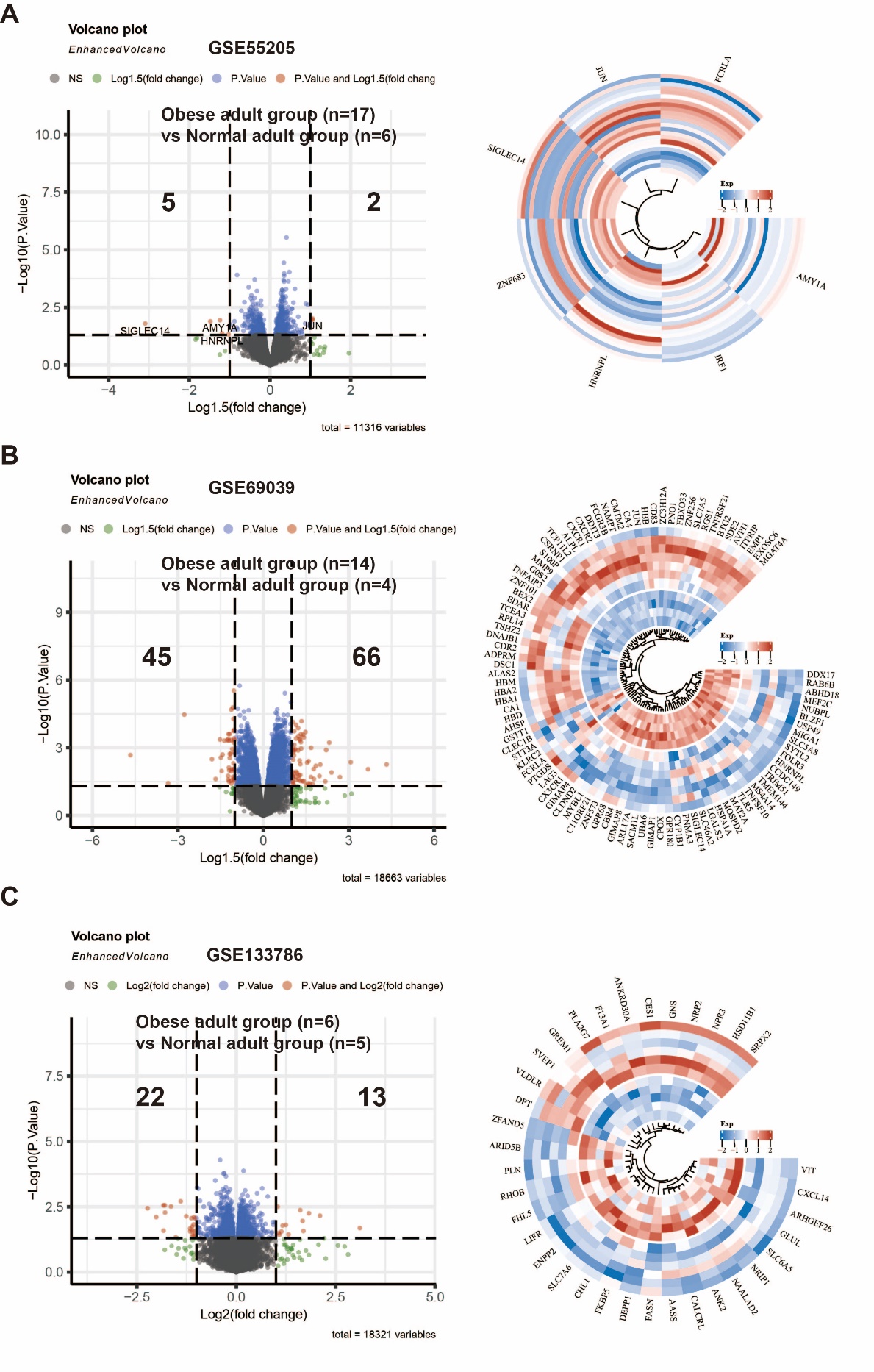


**Supplementary Figure 4 (A, B, C)** Volcano and heat-map of differential genes in **GSE55205, GSE69039 and GSE133786** datasets, respectively.

**Supplementary Figure 5**

**
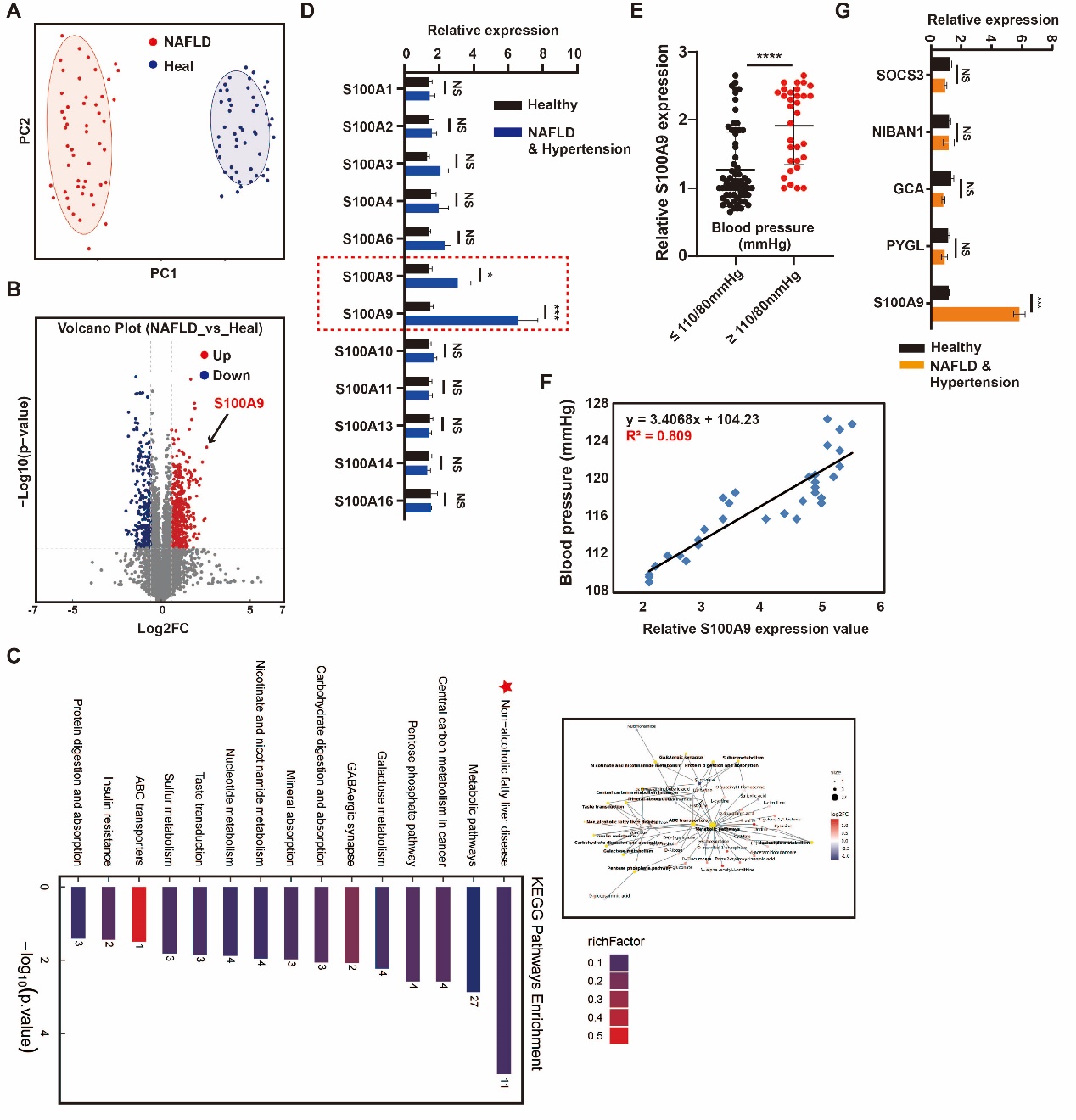
**

**Supplementary Figure 5 (A)** Principal component analysis for sample collection and sequencing; **(B)** Volcano map analysis of sequencing data from obese children collected by Zhoushan Hospital; **(C)** KEGG analyzed the main signaling pathways of differential gene enrichment; **(D)** qPCR detection of differential expression of S100A family genes; **(E)** Analysis of expression difference of S100A9 in obese children with high and low blood pressure groups; **(F)** Correlation analysis of S100A9 expression value and blood pressure in obese children; **(G)** Analysis of the differential expression of five core factors between obese children with hypertension and normal children.

**Supplementary Figure 6**


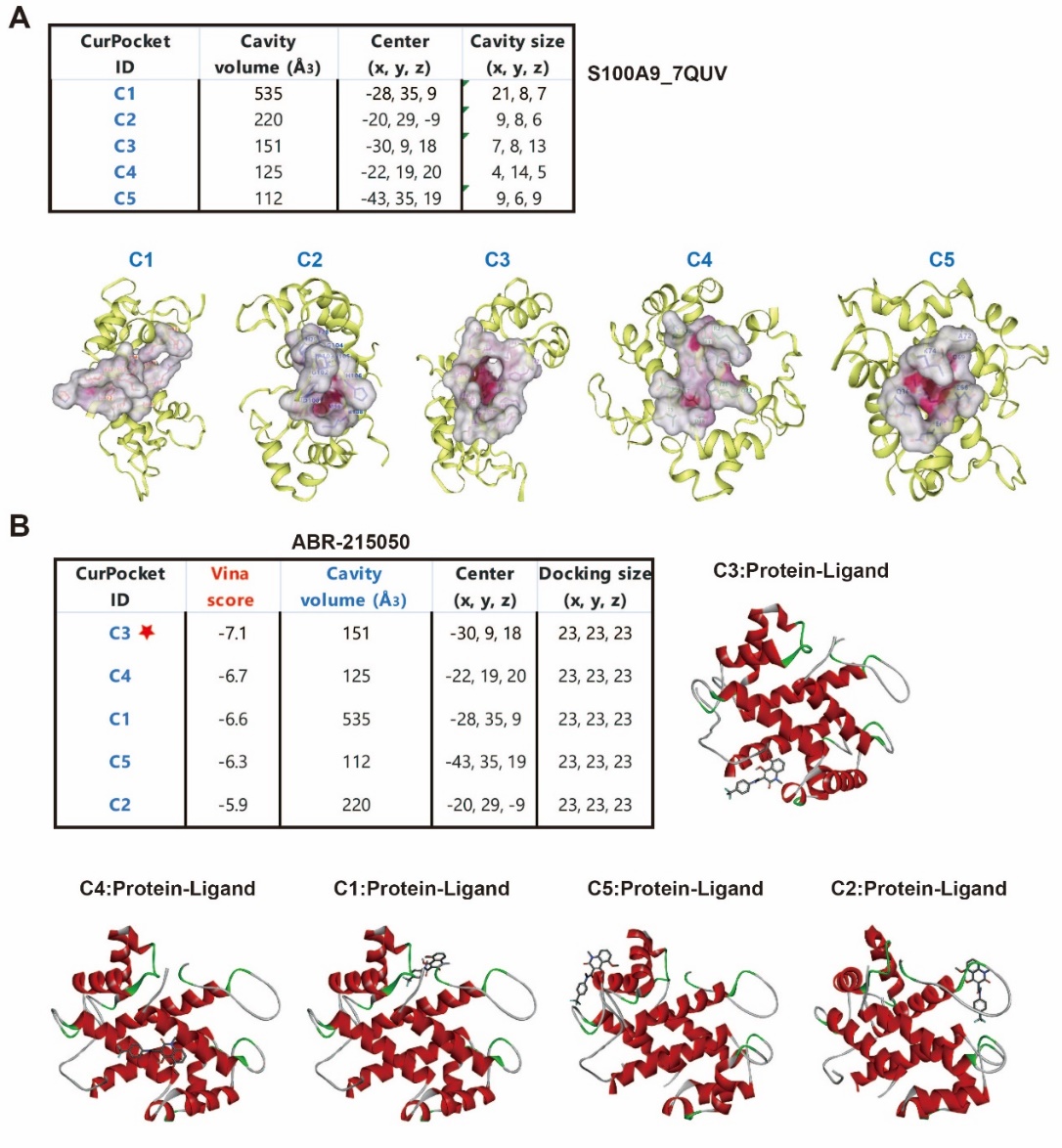


**Supplementary Figure 6 (A)** the pockets of S100A9 protein were analyzed by CB-Dock2; **(B)** The docking analysis results of S100A9 protein and small molecule **ABR-215050** (Vina Score value and conformation of the complex after docking).

**Supplementary Figure 7**

**
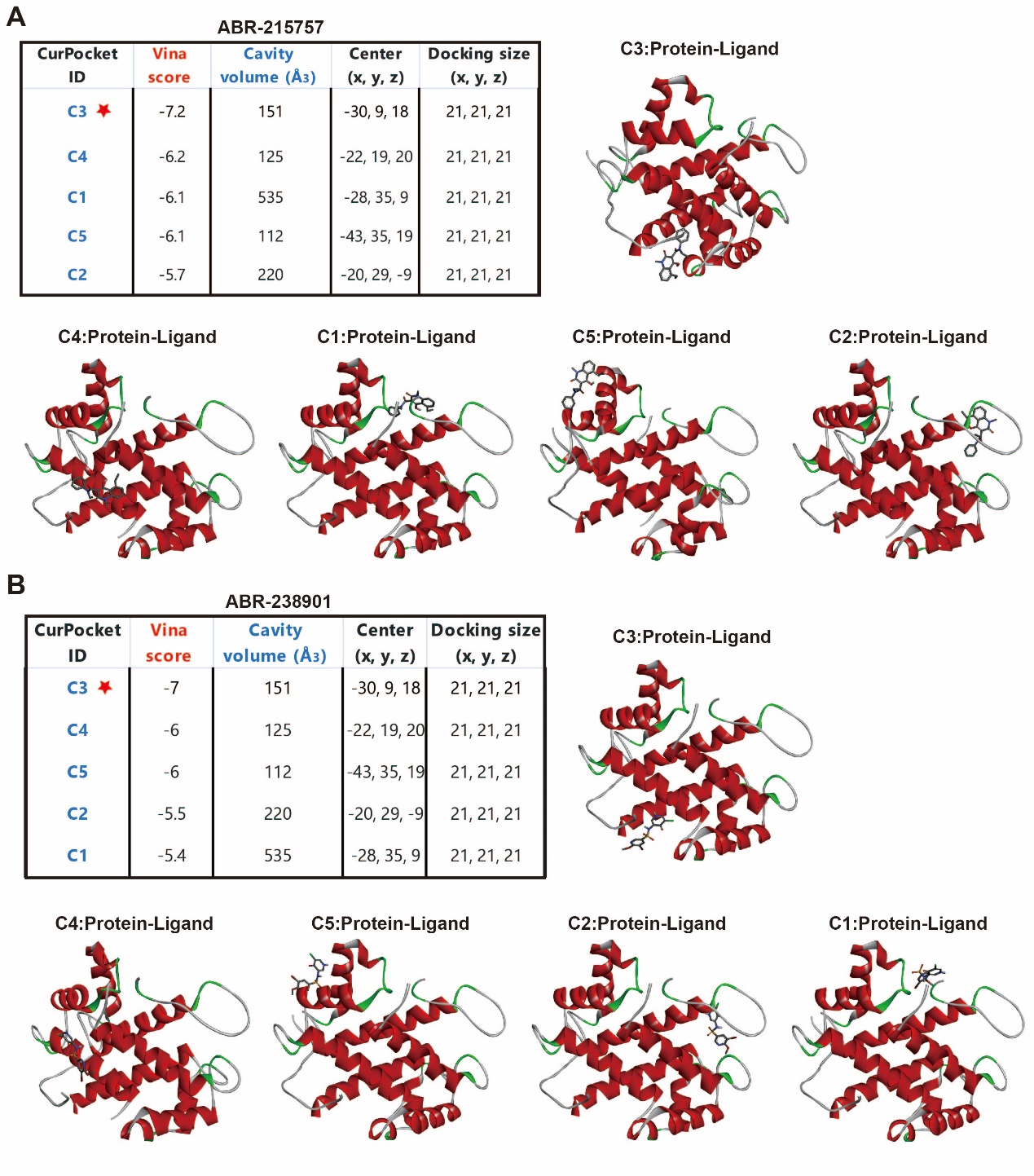
**

**Supplementary Figure 7 (A, B)** the docking analysis results of S100A9 protein and small molecule **ABR-215757** and **ABR-238901** (Vina Score values and conformation of the complex after docking)**,** respectively.

**Supplementary Figure 8**

**
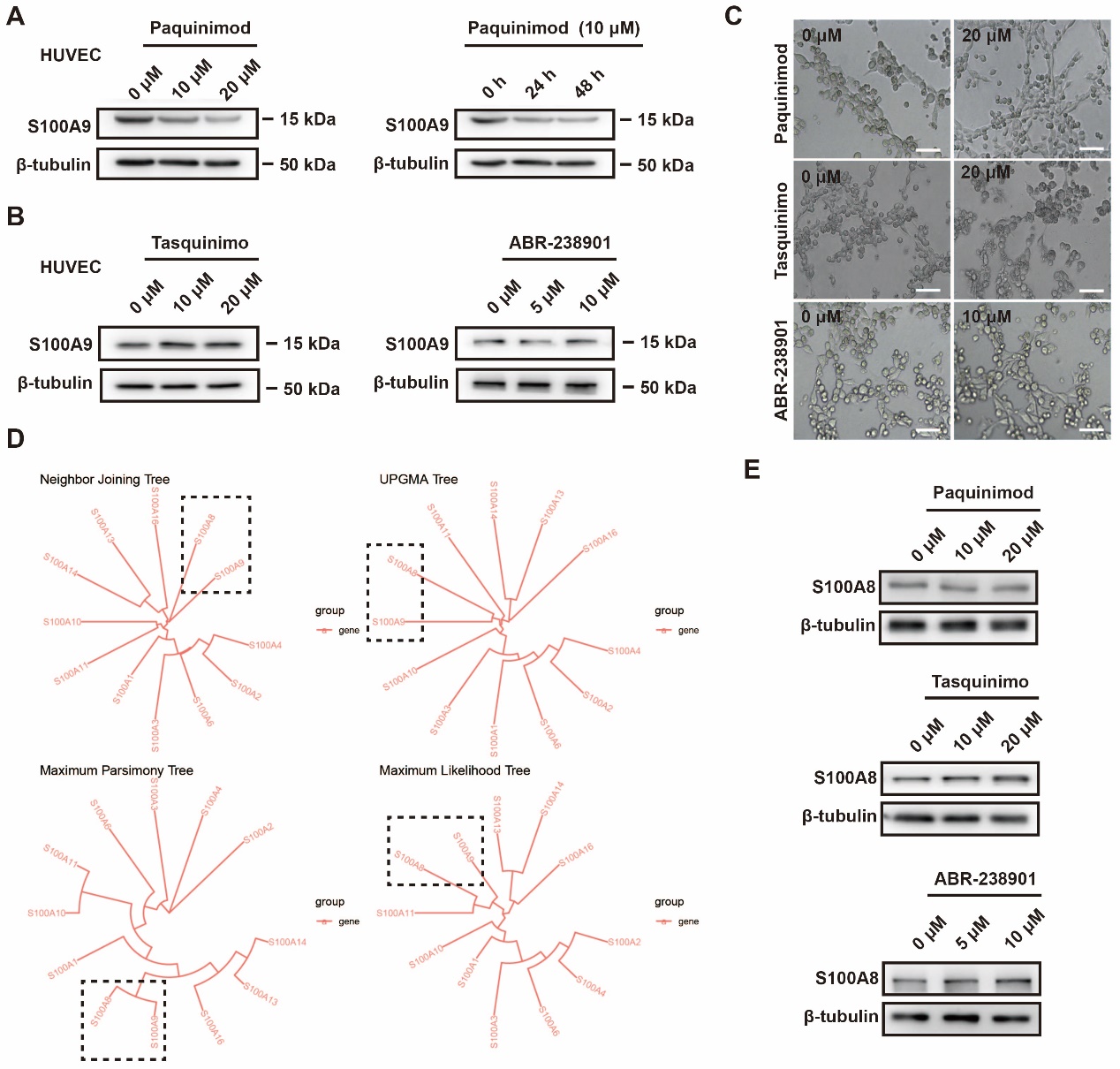
**

**Supplementary Figure 8 (A, B)** the effects of three inhibitors on the protein level of S100A9 were analyzed by WB; **(C)** the effects of these inhibitors on tubular formation in HUVEC cells; **(D)** Analysis of the evolutionary tree of the S100A family; **(E)** The influence of S100A9 inhibitors on the protein level of S100A8.

**Supplementary Figure 9**

**
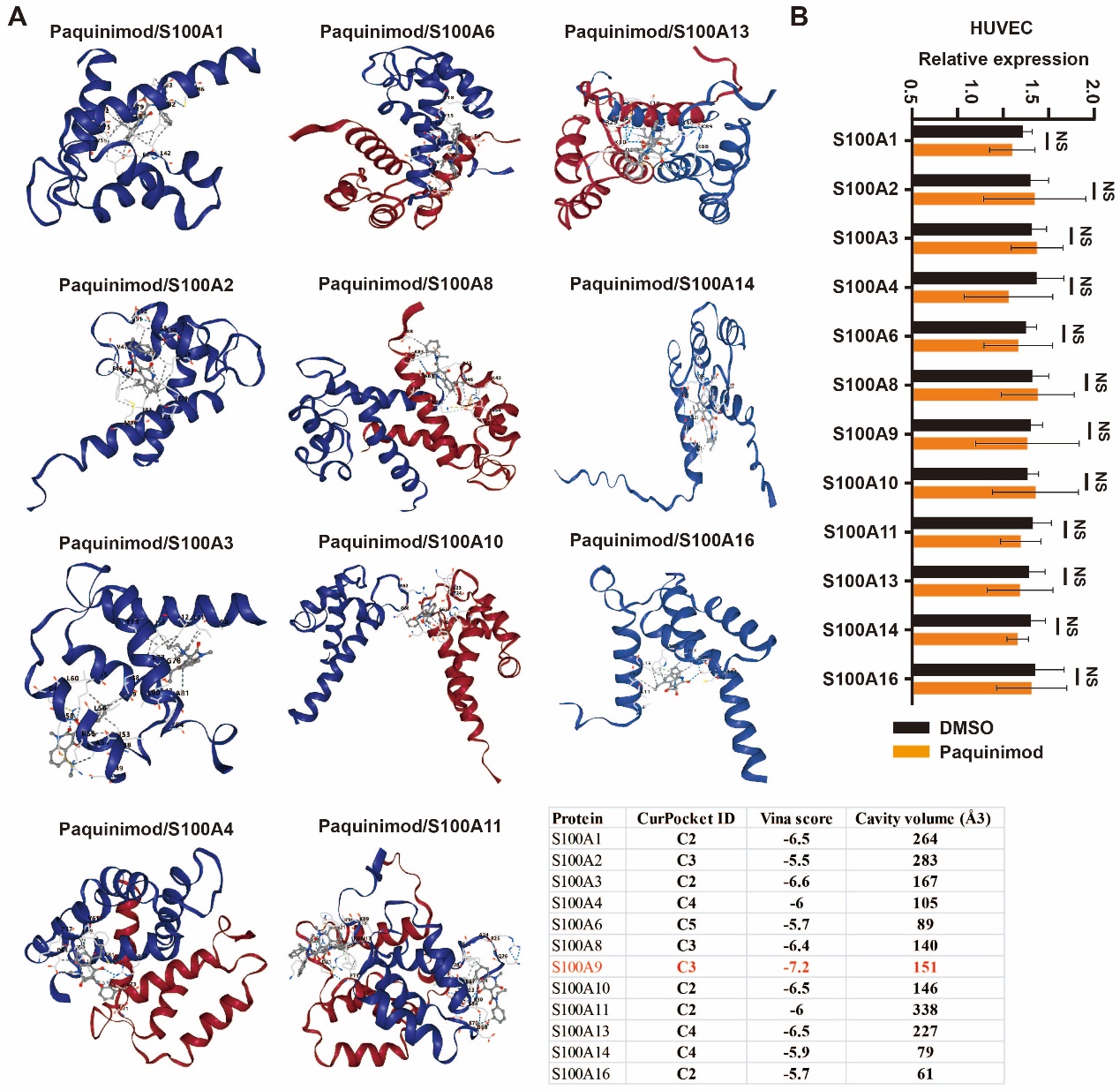
**

**Supplementary Figure 9 (A)** the docking analysis results of S100A family proteins and small molecule Paquinimod; **(B)** The effect of Paquinimod on the mRNA levels of S100A family genes was detected by qPCR.

**Supplementary Method**

***GEO dataset***

**GSE87493**: It includes 12 obese children and 20 healthy children, age range from 3.4 to 17.8 years, median age 13.5 years; **GSE69039**: It includes 14 obese adults and 4 health adults, all of whom are male and aged between 20 to 59; **GSE55205**: It includes 17 obese adults and 6 adults of normal weight, all of whom are male, aged between 20 to 58; **GSE133786**: It includes 6 obese adults and 5 adults of normal weight.

***Identification of the differentially expressed genes***

Gene expression profile files were generated by normalizing the data and correcting the expression value in batches using the "sva" package. The LIMMA package was utilized to identify DEGs between the pediatric sepsis group and the control group, and a volcano plot was produced to highlight the differential expression of DEGs. Adjusted P values were looked at in GEO to account for the possibility of false-positive outcomes. An adjusted P value < 0.05 and |log2FC|> 0.5 were considered to be the cutoffs for DEGs. Using R software's pheatmap package, a heatmap was generated based on the DEGs that had been screened.

***Functional enrichment analysis***

In order to conduct additional functional enrichment analysis, we are currently extracting genes from modules of interest. An examination using Gene Ontology, also known as GO, was carried out so that distinguishing features of the organism’s biological composition could be uncovered. In order to achieve a more in-depth comprehension of the functional characteristics, an investigation into the KEGG pathway enrichment database was carried out.

***Gene-MANIA analysis***

Gene-MANIA imports interaction networks from public databases and predicts these related genes for a query gene set. It integrates with Cytoscape for network visualization, analysis and automation. Gene-MANIA will find these genes that may share functions with it based on their interactions with it, and analyze and predict the functions of those genes. The gene network involved in this study consists of five genes: SOCS3, NIBAN1, GCA, PYGL and S100A9.

***Molecular docking*** (https://cadd.labshare.cn/cb-dock2/php/index.php)

Molecular docking is carried out using CB-Dock2. The CB-Dock2 is an improved version of the CB-Dock server for protein-ligand blind docking, integrating cavity detection, docking and homologous template fitting. Given the three- dimensional (3D) structure of a protein and a ligand, we can predict their binding sites and affinity for computer-aided drug discovery.

***WGCNA analysis and module identification***

The WGCNA method augments the analysis of gene set expression. The WGCNA R package is employed to construct and modularize distinct gene networks across various stages. At first, the Pearson's correlation matrices and average linkage method were both performed for all pair-wise Genes,Then, a weighted adjacency matrix was constructed using a power function A_mn= |C_mn|^β (C_mn = Pearson's correlation between Gene_m and Gene_n; A_mn = adjacency between Gene m and Gene n). β was a soft-thresholding parameter that could emphasize strong correlations between Genes and penalize weak correlations. After choosing the power of 26, the adjacency was transformed into a topological overlap matrix (TOM), which could measure the network connectivity of a Gene defined as the sum of its adjacency with all other Genes for network Gene ration,and the corresponding dissimilarity (1-TOM) was calculated. To classify Genes with similar expression profiles into Gene modules,average linkage hierarchical clustering was conducted according to the TOM-based dissimilarity measure with a minimum size (Gene group) of 30 for the Genes dendrogram.

A cluster analysis is conducted on these samples to identify significant outliers. An automated network system is utilized to establish co-expression networks. These modules undergo functional evaluation through hierarchical clustering and dynamic tree cutting. The Module Membership (MM) and Gene Significance (GS) are assessed to correlate modules with clinical attributes. Modules displaying the highest Pearson Module Membership correlation (MM) and a p-value of 0.05 are classified as central modules. An MM value surpassing 0.8, coupled with a GS exceeding 0.1, indicates that the module demonstrates strong connectivity and clinical relevance. Gene information related to the pertinent modules is provided to promote further investigation.

Genes within the clinically significant gene module network with a GS value exceeding 0.2 and an MM value greater than 0.8 are categorized as hub genes. Genes identified as overlapping are selected as candidates for crucial roles. Venn R package is employed to generate significant gene diagrams.

**Bioinformatics analysis**

All bioinformatics analyses were conducted on the online website "SangerBox **^[1]^**", including correlation analysis tools, functional analysis and WGCNA analysis. http://sangerbox.com/

***RNA extraction, reverse transcription and RT-qPCR***

Total RNA was isolated using a RNeasy kit (Vazyme, Nanjing). 1μg of total RNA was used for first strand cDNA synthesis with SuperScript II Reverse Transcriptase and oligo-dT primers (Invitrogen). Real-time PCR was performed with SYBR® Green PCR Master Mix (Vazyme, Nanjing) using Quantitect primers in a LightCycler® 480 II Detection System (Roche). Relative amount of all mRNAs was calculated using the comparative CT method after normalization to β-actin.

VEGF: Forward Primer: 5’- AGGGCAGAATCATCACGAAGT -3’;

Reverse Primer: 5’- AGGGTCTCGATTGGATGGCA -3’

VCAM-1: Forward Primer: 5’- TTTGACAGGCTGGAGATAGACT -3’;

Reverse Primer: 5’- TCAATGTGTAATTTAGCTCGGCA -3’

***Three-dimensional culture assay (tube formation)***

HUVEC cells were treated with Paquinimod for 24 hours, subsequently seeded into a 24-well plate pre-coated with matrigel, and cultured in 250 μL of RPMI-1640 supplemented with 10% FBS for an additional 24 h. Tube structures were imaged using a light microscope and evaluated to assess their formation capacity.

***ROS level measurement***

The determination of ROS in cells was carried out using DCFH-DA (S0033S, Beyotime), in accordance with the manufacturer's instructions. In short, after adding the inhibitor for a period of time, 10 μM DCFH-DA was loaded for 30 minutes. The cells were washed three times with frozen PBS and immediately observed under a fluorescence microscope, and the fluorescence differences were analyzed using a flow cytometer.

***Data collection of obese children in Zhoushan city Area***

***(1) On-site implementation and sample collection:***

A research site was established at the Zhoushan Hospital & Zhoushan Women and Children Hospital to include overweight or obese children who met the criteria. Fasting liver biochemistry, blood glucose and insulin data of the research subjects were collected, and 5mL of fasting venous blood was collected.

***(2) Insulin detection:*** ELISA is used to detect fasting insulin levels.

***(3) Blood glucose test:*** Measure the fasting blood glucose level.

***(4)* Research subjects**: Obese children who visited Hospital were included.

The specific inclusion and exclusion criteria are as follows:

**Inclusion criteria:**

1) Age: 6 to 18 years old;

2) BMI meets the diagnostic criteria for being overweight or obese.

**Exclusion criteria:**

1) Those with secondary obesity caused by genetic metabolic diseases, endocrine diseases, etc.

2) Patients with liver-related diseases such as viral liver diseases, alcoholic liver diseases, and autoimmune liver diseases;

3) Those with a long-term history of smoking and drinking;

4) Those with malignant tumors or severe organ damage.

***(5) Diagnostic criteria for overweight or obesity***: Diagnostic criteria for overweight or obesity in children aged 6 to 18 are based on "WS/T 586-2018 Screening for Overweight and Obesity in School-Age Children and Adolescents".

***(6) Sample size***: Based on the existing work of our team, approximately 40% to 50% of the obese children with NAFLD have hypertension. Therefore, we measured the blood pressure of 100 children who met the NAFLD criteria and selected the top 50 with higher blood pressure as the experimental group.

***(7)* Epidemiological investigation**: Basic sociodemographic information (age, gender, family- related situation, etc.), health status and lifestyle behavior patterns (diet, exercise, sleep status, etc.) of the study subjects were collected through the "Zhoushan Obese Children Epidemiological Investigation Questionnaire".

**Supplementary table**

| **Inhibitor** | **Alias** | **Character** | **Relevant research** |
| --- | --- | --- | --- |
| Paquinimod | ABR-215757 | Specific inhibitors of S100A8/S100A9 with oral activity | 1. Paquinimod is an immunomodulatory compound that can prevent S100A9 from binding to TLR-4. Prophylactic treatment with the S100A9 inhibitor Paquinimod can reduce the pathology of experimental collagenase-induced osteoarthritis. 2. Paquinimod is an effective inhibitor of the development of pancreatitis and diabetes in NOD mice. |
| Tasquinimo | ABR-215050 | S100A9 inhibitor | Tasquinimod is an orally active quinoline-3-formamide that has a high affinity for HDAC4 and S100A9 in cancer and infiltrates host cells in the damaged tumor microenvironment, inhibiting the adaptive survival pathways required for angiogenic responses |
| ABR-238901 | ABR-238901 | An orally effective S100A8/A9 blocker that can inhibit the interaction between S100A8/A9 and its receptors RAGE and TLR4 | ABR-238901 leads to a reduction in angiogenesis and a decrease in IL6 and IL10 in MDSC |

**References**

1. Weitao Shen, Ziguang Song, Xiao Zhong, et al. Sangerbox: A comprehensive, interaction-friendly clinical bioinformatics analysis platform. *iMeta* 2022;1: e36.

https://doi.org/10.1002/imt2.36.
